# Descriptive epidemiology of SARS‐CoV‐2 Beta (B.1.351) variant cases in England, December 2020 to June 2022

**DOI:** 10.1111/irv.13204

**Published:** 2023-11-15

**Authors:** Mary A. Sinnathamby, Asad Zaidi, Katherine A. Twohig, Shirin Aliabadi, Nurin Abdul Aziz, Natalie Groves, Eileen Gallagher, Simon Thelwall, Gavin Dabrera

**Affiliations:** ^1^ COVID‐19 Vaccines and Epidemiology Division, Public Health Programmes Directorate UK Health Security Agency London UK; ^2^ Genomics Public Health Analysis, TARZET Division, Clinical and Emerging Infections Directorate UK Health Security Agency London UK

**Keywords:** B.1.351, England, SARS‐CoV‐2 Beta, second generation surveillance system, travel, whole genome sequencing

## Abstract

The emergence of the SARS‐CoV‐2 Beta (B.1.351) variant in November 2020 raised concerns of increased transmissibility and severity. We describe the epidemiology of 949 confirmed SARS‐CoV‐2 Beta variant cases in England, identified between December 2020 and June 2022. Most cases were detected in the first 3 months. A total of 10 deaths (1.1%; 10/949) were identified among all cases and of those with travel information, 38 (4.9%; 38/781) cases with hospital admissions within 14 days of a positive test being detected. 52.9% (413/781) cases were imported. This study reinforces the importance of monitoring of travel‐associated cases to inform public health response and reduce transmissibility of new variants.

## 1 BACKGROUND

South Africa saw the emergence of a new, rapidly spreading variant of SARS‐CoV‐2, B.1.351, also known as Beta, in three of its provinces in early November 2020. The variant has since been confirmed to have been imported to several countries worldwide.^1^ The SARS‐CoV‐2 Beta variant was designated as a variant of concern on 18 December 2020 by WHO due to concerns around increased transmissibility and severity as well as reduced vaccine protection as experienced in many countries shortly after this designation.^1^, ^2^, ^3^, ^4^, ^5^ Phylogenetic information of early cases of SARS‐CoV‐2 B1.351 in England have been described previously elsewhere.^6^

In England, several public health and social measures were implemented during the winter of 2020/2021, due to a surge of COVID‐19 cases.^7^ These included travel bans from different countries where the prevalence of this variant was seen to be increasing.^8^

Following recent authorisation, a vaccine based on a recombinant spike protein from the Beta variant (VidPrevtyn Beta®) is being used in a national booster campaign for specific risk groups. In this context, we describe the epidemiology of whole genome sequencing (WGS)‐confirmed SARS‐CoV‐2 B.1.351 cases in England, with a particular focus on travel status and hospitalisations, from its first detection in December 2020 through to June 2022.

## 2 METHODS

Polymerase chain reaction (PCR)‐confirmed SARS‐CoV‐2 cases in England with specimen dates between 1 December 2021 and 30 June 2022 were extracted from UKHSA's second generation surveillance system (SGSS).^9^ Cases with validated WGS results were abstracted from linkage to data processed from the Cloud Infrastructure for Big Data Microbial Bioinformatics (CLIMB) database.^10^ There was an estimated reporting lag of at least 2 weeks between an individual's SARS‐CoV‐2 sample collection and WGS results becoming available.^11^ Information on inclusion criteria for PCR testing and WGS of cases and Beta variant cases have been described previously.^7^, ^9^

WGS‐confirmed Beta variant cases were further linked to NHS Digital's Emergency Care Data Set (ECDS) and Secondary Uses Service (SUS) datasets for hospitalisation information, where a case was defined as having been admitted to hospital where the specimen dates are 14 days before the admission date to 1 day after.^12^ Vaccination status of WGS‐confirmed Beta variant cases was derived from linkage to the National Immunisation Management System (NIMS) to determine a case's vaccination status 14 days prior to their positive specimen date.^13^ WGS‐confirmed Beta variant cases were also linked to UKHSA's COVID‐19 mortality dataset for COVID‐19 specific deaths, where a COVID‐19 specific death was defined as a death reported to have occurred within 28 days of a positive COVID‐19 test.^14^

Travel status was identified through UKHSA's COVID‐19 Integrated Travel dataset, which combines travel information from Passenger Locator Forms (PLFs), NHS Test & Trace's Contact Tracing and Advice Service (CTAS), SGSS and from interviews with Health Protection Teams across England.^15^, ^16^ Valid travel statuses included confirmed travel (imported cases), contacts of travellers (secondary cases), cases confirmed to have no travel history (sporadic cases) and unknown travel. Information on country of travel for imported cases were categorised in accordance with WHO regions with further categorisation of the African region into Eastern and Southern regions.^17^ Cases who travelled to more than one country were categorised as multiple countries of travel. Information on international travel restrictions for England was collated from official sources.^8^

Imported cases were defined as WGS‐confirmed cases with a travel history, secondary cases as those who had been a contact of an imported case and sporadic cases as those with no travel history nor contact with an imported case.

Imported Beta variant cases were linked to a list of Managed Quarantine Facilities (MQFs). MQFs were sites where individuals travelling to the UK from a country on the travel ban list were required to isolate for 10 days. This policy was introduced from 15 February 2021 as a mitigation measure against the transmission of COVID‐19 and SARS‐CoV‐2 variants.^18^, ^19^

Descriptive analyses were also categorised by travel status and assessed by age group, sex, UKHSA region, and ethnicity.

## 3 RESULTS

Between 1 December 2020 and 30 June 2022, a total of 949 confirmed SARS‐CoV‐2 Beta cases, referred to as cases hereafter, were identified in England through WGS.

Travel data were available for 781 (82.3%) cases. Of these, 413 (52.9%) cases were imported, whereas 368 (47.1%) cases were as secondary or sporadic (Table 1). A total of 168 (18.0%) cases were classified as having an unknown travel status.

**TABLE 1:** Demographic description of confirmed SARS‐CoV‐2 Beta (B.1.351) cases in England by travel status, up to 30 June 2022.

	Imported cases (*n* = 413)		Secondary/sporadic cases (*n* = 368)		Total (*n* = 781)	
	n	%	n	%	n	%
Age						
<20	40	9.7%	90	24.5%	130	16.6%
20–29	100	24.2%	71	19.3%	171	21.9%
30–39	88	21.3%	79	21.5%	167	21.4%
40–49	92	22.3%	53	14.4%	145	18.6%
50–59	51	12.3%	30	8.2%	81	10.4%
60–69	30	7.3%	25	6.8%	55	7.0%
70–79	9	2.2%	11	3.0%	20	2.6%
80+	2	0.5%	7	1.9%	9	1.2%
Unknown	1	0.2%	2	0.5%	3	0.4%
Sex						
Male	231	55.9%	200	54.3%	431	55.2%
Female	179	43.3%	164	44.6%	343	43.9%
Unknown	3	0.7%	4	1.1%	7	0.9%
Ethnicity						
White	78	18.9%	140	38.0%	218	27.9%
Asian/Asian British	155	37.5%	101	27.4%	256	32.8%
Black/African /Caribbean/Black British	95	23.0%	67	18.2%	162	20.7%
Mixed/Multiple ethnic groups	9	2.2%	8	2.2%	17	2.2%
Other	21	5.1%	4	1.1%	25	3.2%
Unknown	55	13.3%	48	13.0%	103	13.2%
Hospitalisations						
Admitted to hospital^a^	19	4.6%	19	5.2%	38	4.9%
No hospital admission	394	95.4%	349	94.8%	743	95.1%
Death within 28 days after positive test^b^						
Yes	2	0.5%	5	1.4%	7	0.9%
No	411	99.5%	363	98.6%	774	99.1%
Vaccinated (≥14 days prior to positive test date)						
Yes	23	5.6%	60	16.3%	83	10.6%
No	278	67.3%	249	67.7%	527	67.5%
Unknown/unlinked^c^	112	27.1%	59	16.0%	171	21.9%
UKHSA regions						
East Midlands	26	6.3%	12	3.3%	38	4.9%
East of England	46	11.1%	29	7.9%	75	9.6%
London	174	42.1%	175	47.6%	349	44.7%
North East	7	1.7%	16	4.3%	23	2.9%
North West	26	6.3%	43	11.7%	69	8.8%
South East	67	16.2%	33	9.0%	100	12.8%
South West	15	3.6%	9	2.4%	24	3.1%
West Midlands	27	6.5%	31	8.4%	58	7.4%
Yorkshire and Humber	21	5.1%	12	3.3%	33	4.2%
Unknown	4	1.0%	8	2.2%	12	1.5%
Regions of travel						
Americas	3	0.7%				
Eastern Africa	27	6.5%				
Southern Africa	64	15.5%				
Eastern Mediterranean	55	13.3%				
South East Asia	60	14.5%				
Europe	18	4.4%				
Western Pacific	7	1.7%				
Multiple countries	179	43.3%				

^a^ Cases admitted to hospital where the specimen dates are 14 days before the admission date to 1 day after.

^b^ Three cases with an unknown travel status also died.

^c^ Cases that were unable to be linked to NIMS.

**FIGURE 1:**
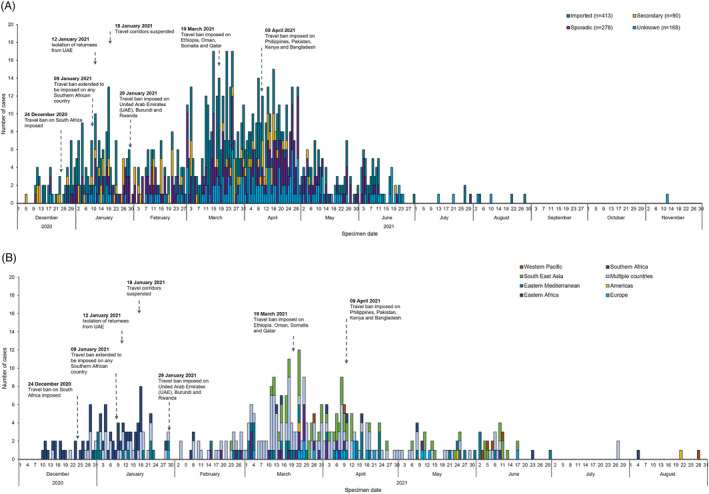
Epidemic curve of SARS‐CoV‐2 Beta (B.1.351) cases (*n* = 949) by (A) travel status and (B) regions of travel for imported cases (*n* = 413) and imposed restrictions in England.

The distribution of cases varied across the study period with most cases, particularly imported cases, detected prior to April 2021 (Figure 1A). The largest count of cases per day in the initial wave was noted on 18 January 2021 (*n* = 17) approximately 2 weeks after the imposition of travel bans on the Southern African region with cases decreasing thereafter (Figure 1A and B). A second spike in the count of cases per day was on 25 March 2021 (*n* = 20) with further decreases observed thereafter following additional travel bans imposed mostly for countries of the Eastern Mediterranean and South East Asia regions (Figure 1B, Table 1).

The majority of imported cases had a travel history to multiple countries (43.2%) (Figure 1B, Table 1), followed by single region of travel as Southern Africa (15.5%), South East Asia (14.5%) and Eastern Mediterranean (13.3%) (Table 1).

Just over 50% of both imported and secondary/sporadic cases were male. Among imported cases, the most common age groups were the 20–29 year age group (24.2%) and the 40–49 year age group (22.3%). In contrast, among secondary/sporadic cases, <20 year age group comprised the highest proportion (24.5%) followed by the 30 to 39 year age group (21.5%).

Nearly half of imported and secondary/sporadic cases were allocated to the London region (42.0% and 47.6%, respectively; Table 1). However, the second most common region was the North West for secondary/sporadic cases and the South East for imported cases.

By ethnicity, the largest groups of imported cases were Asian (37.5%) and Black (23.0%). In comparison, secondary/sporadic cases were mainly White (38.0%) and Asian ethnicity (27.4%) (Table 1).

By COVID‐19 vaccination status, only a small proportion of cases (5.6% of imported cases and 16.3% of secondary/sporadic cases) were identified to have been vaccinated 14 days or more prior to their positive specimen date (Table 1).

A total of 10 deaths (1.1% of all cases) occurred during the study period and consisted of two imported cases, five secondary/sporadic cases and three cases with unknown travel status. A total of 38 cases were admitted to hospital within 14 days of a positive test. There was little difference in the risk of hospitalisation by travel status with 5.2% of secondary/sporadic cases being admitted to hospital compared to 4.6% of imported cases.

Of the 413 imported cases, 300 cases were identified to have a specimen date on or after the implementation of MQF requirements (15 February 2021). Of these 300 cases, 114 cases were identified to have become cases after their respective country of travel had been added on the travel ban countries list. Of these 114, 62.0% (71/114) had information related to MQF use.

## 4 DISCUSSION

This study indicates that the overall detection of the SARS‐CoV‐2 Beta variant was low in England, despite concerns over increased transmissibility and potential adverse effect on the efficacy of COVID‐19 vaccines.^1^, ^4^ Between December 2020 and March 2021, detected cases of the Beta variant were largely among those who had travelled internationally. From April to November 2021, the dominance of other SARS‐CoV‐2 variants, such as Delta and Omicron variants in England, likely played a role in the suppression or reduced circulation of the Beta variant.^20^, ^21^

In South Africa, where the Beta variant originated, the variant caused the nation's largest recorded wave of COVID‐19 up until that time. Increased mortality and hospitalisation rates were also observed.^1^ A study in Qatar also found increased odds of severe disease and death among cases with the Beta variant compared to cases with the Alpha variant.^4^ In contrast, this study highlighted low numbers of Beta variant cases, and associated deaths and hospitalisations in England.

These differences may be attributable to the role of public health and social measures introduced in response to the rise of the Beta variant in England. Nearly half of all detected cases were imported from high‐prevalence countries and approximately 20% of all imported cases occurred within managed quarantine facilities, breaking chains of onward transmission that could have led to widespread community transmission.^22^

It is important to note the impact of the lag in obtaining sequencing results and the varying sequencing coverage during the study period. Sequencing coverage increased across the study period reaching around 50% in the latter periods, which highlights that case ascertainment may have improved during the study period.^11^ Additionally, the identification of MQF use was limited by the availability of separate testing information, which was required for data linkage; improvements of such datasets would support future pandemic responses.

This study is also relevant at this current time as it provides insight into transmission dynamics and severity measures in light of ongoing discussions on the consideration of including the Beta variant as a vaccine candidate for booster SARS‐CoV‐2 vaccines.^23^

This study reinforces the importance of establishing surveillance systems to detect and monitor cases associated with travel, secondary transmission and sporadic detection to inform prompt public health action for response and reduce the spread of new and emerging SARS‐CoV‐2 variants.

## AUTHOR CONTRIBUTIONS

**Mary Sinnathamby**: Conceptualization (equal); data curation (lead); formal analysis (lead); methodology (equal); writing—original draft (lead). **Asad Zaidi**: Data curation (supporting); writing—review and editing (equal). **Katherine Twohig**: Writing—review and editing (equal). **Shirin Aliabadi**: Writing—review and editing (equal). **Nurin Abdul Aziz**: Writing—review and editing (equal). **Natalie Groves**: Writing—review and editing (equal). **Eileen Gallagher**: Writing—review and editing (equal). **Simon Thelwall**: Writing—review and editing (equal). **Gavin Dabrera**: Conceptualization (equal); methodology, (equal); Writing—review and editing (equal).

## CONFLICT OF INTEREST STATEMENT

Dr. Dabrera reports the predecessor of the organisation he works for, Public Health England (PHE), received an unrestricted grant from GSK to undertake a study on the outcome of patients who received parenteral zanamavir. The funder received data and interim reports from PHE but did not influence analysis and reporting of the study. I Gavin Dabrera had no involvement in the GSK‐funded study on parenteral zanamavir. Furthermore, the currently submitted work was part of the public health response activities to COVID‐19 and had no relationship to GSK nor the study on parenteral zanamavir. No conflicts of interest declared for all other authors.

## Data Availability

Data cannot be made publicly available for ethical and legal reasons, that is, public availability would compromise patient confidentiality as data tables list single counts of individuals rather than aggregated data.
